# The Heatonian Method for the Radical Cure of Hernia

**Published:** 1881-04

**Authors:** W. H. Heath

**Affiliations:** Assistant Surgeon U. S. Marine Hospital Service


### THE HEATONIAN METHOD FOR THE RADICAL CURE OF HERNIA.

BY W. H. HEATH, M. D., ASSISTANT SURGEON U. S. MARINE HOSPITAL SERVICE.

THE radical cure of hernia has attracted the attention of surgeons for ages fully as much as any surgical procedure that I know of, but without the success the importance of the measure or the labor expended would lead us to hope. The numerous methods, at times popular, have all justly fallen into disfavor, and at the present time I know of none of the older operations, such as Wutzer's, Wood's or Agnew's, which a sound surgeon has much if any confidence in or would attempt without reluctance ; and in consequence of the want of success following and the attending dangers, they have been practically abandoned.

Now it is to the almost complete absence of danger, the great encouragement held out as to results, and its sound pathology, that the Heatonian method has in my opinion more claims upon the attention of surgeons than all others, and more than it has yet received. I am not prepared to say definitely how much favor it has been accorded, but my observations and inquiries lead me to think that it has not been given the trial its merits deserve, and here in Buffalo I doubt if the operation has been resorted to many times, if at all, by any one but myself. It is, however, a comparatively recent method ; up to four years ago it was not in the hands of the profession, and since that time, whether from distrust of any measure claiming so much and apparently so simple, or from some other cause, but little has been done with the operation anywhere. In this connection it may be well to allude to the fact that Dr. Heaton was for years ostracised from the regular profession, and looked upon with little confidence, in consequence of his keeping his method secret. Dr. W. B. DeGarmo, in the *N. Y. Medical Record* of February 7, 1880, in speaking of this operation, mentions this matter and states that his action was in consequence of the very indifferent manner in which some eminent medical men of Boston treated his invitation to come and see him operate, causing him to retain that which it was his intention to have given to the profession.

Be this as it may, neither its simplicity nor any prejudice against the author of it, if any exists, are sufficient to condemn it without a fair trial, more especially as it is an operation of no severity and almost devoid of danger.

The article of Dr. De Garmo referred to will be found as interesting as the ingenious instrument for operating upon these cases is valuable, and to which he calls attention in the same place. I have used this needle with such satisfaction that I cannot but call attention to my experience with it.

The older operations are all based upon the principle of obliterating the hernial passages by inflammatory adhesions in the sac wall, or plugging with invaginated tissues, and are necessarily operations of magnitude or severity, differing entirely from the Heatonian method, wherein any degree of inflammation worthy the name is antagonistic, in fact detrimental to the result, and the sac takes no part whatever in effecting the cure. In this alone, therefore, the operation ceases to be of a formidable character.

The pathology of it, according to the author, consists in developing by the action of the irritant, which is also an astringent, a tendinous irritation, causing a contraction of the fibrous tissues and rings, which the circular arrangement of fibres makes possible, and the formation of strongly plastic lymph. Recognizing that the fibrous structures and rings are the structures primarily and principally in fault, to these alone is the remedy addressed. The mild character of the irritant, the operation being subcutaneous, the parts so slightly vascular, being nourished by nutritive juices, are the reasons given for the irritation exceeding no further bounds, while the permanency of the effect produced is due to the interstitial and hyperplastic changes and the disposition of fibrous tissues generally to recover slowly, analogy being drawn to the duration of changes in similar structures elsewhere, as around joints and the heart valves. The irritant used is the fluid extract of Quercus Alba prepared in vacuo, to which is added the Solid Extract in the proportion of 14 grains to half an ounce and a little morphia to lessen pain; this is triturated with heat until a very perfect solution is obtained. The instrument with which the operation can most satisfactorily be done is of Dr. De Garmo's device, made by Tiemann & Co., N. Y., and described as stated in the *Medical Record.* It is a 20-m syringe, screw-piston to gradually deposit its contents, and a trocar needle by which its point is guarded while in the inguinal canal. The same irritant in different proportion, the Solid Extract being reduced to the consistency of paste by the fluid, with a modified instrument for its introduction is also advised by the author, but more particularly to old and large hernia, where the apertures are patulous, and in those cases where the milder one does not have the desired effect, the paste being more easily handled and the irritating property more enduring.

The patient, whose bowels should be moved by oil the day previous, is to be placed in bed and the hernia and sac, if possible, reduced. The presence of the sac does not prevent a successful result, it merely diminishes the effect. The operation consists in locating the exact position of the external abdominal ring, by invaginating a finger of the right hand in the scrotum and fixing its position on the exterior by a finger of the other hand, which is made to press directly down upon it, or, if possible, in it. The instrument, already prepared, is carried with a sharp thrust quickly through the integument, just passing the external pillar, the needle then guarded, is carried on into the canal; care being exercised not to injure the cord, or penetrate into the peritonial cavity. The position of the beak of the instrument should at this stage be confirmed by a finger again invaginated through the scrotum, and the irritant deposited as it is withdrawn, all the fibrous strictures being wet. A

bandage and compress, previously applied, are then carefully adjusted into position and so arranged as to press with considerable firmness downwards and upwards in the direction of the canal, with somewhat less pressure over the internal than the external ring. This procedure is not accompanied by much pain, and that which follows is of short duration and but moderate intensity; tenderness exists in a degree for some little time, but not enough to require the compress to be removed, or to produce any inconvenience. The recumbent position for a week or so, and no movement from the bowels, are to be insisted on, for the protrusion must not be allowed to descend after its reduction, and the irritant deposited. The bandage should be worn or a light truss applied for a month or more, as a precaution, but after that it may be discontinued and the case considered cured. In a certain number of cases the operation has to be repeated, more especially where the apertures are large and patulous, or the cause has been violence tearing the fibrous rings, and in congenital hernia where they apparently are deficient in fibrous structure.

Simple as all this appears, it requires considerable care and dexterity; the cord, which must be pushed aside, may be displaced and in part overlie the sac, which may itself be irreducible. The direction of the canal and position oi internal ring changed, the possibility of transfixing one of the pillars, wounding the cord, or entering the abdominal cavity, are all to be remembered and avoided. The attention to every detail in operating, adjusting the compress and bandage, and the after-care are so important as to largely determine the result in most cases. An hour or so, therefore, in the dissecting room, with a long needle, would not be mis-spent, but would aid to familiarize a beginner with the points most important to find, or as far as possible, avoid.

This method I have resorted to 12 times with one failure (I believe due entirely to a nurse's carelessness) and one accident where I deposited the irritant in the areola tissue of the cord, which from pressure of the hernia had been spread out and displaced, almost beyond recognition. Nine of the cases I consider permanently cured, and two are yet under observation in my wards. All the cases were of the oblique reducible inguinal variety, 8 of 5 years standing, 1 of 17 years, 1 of 12 years, 2 over 10 years.

In no case did I observe a single bad symptom, elevation of temperature or pulse rate, and but little, if any, of what may properly be termed suffering, and with the exception mentioned every case left my hands, after keeping them as long as I could, apparently cured. I say apparently cured because the standing argument against the permanency of the result at once is raised, and I cannot say positively, beyond preadventure, they are permanently cured, for they are beyond my observation now. Two of the men I had the good fortune to see and examine some six months after, and in both the inguinal canal was closed perfectly, and the protrusion had never appeared since leaving the hospital. One of them had subjected his case to a pretty severe test, having worked as coal heaver on a southern steamer ever since. I do not recall what kind of work the other had been engaged in, but as he was an ordinary sailor, I do not doubt the radical care was strongly tested.

Dr. De Garmo, in the article referred to, and whose experience no doubt has been extensive, speaks of the frequency of cases completely cured by Dr. Heaton, which have fallen to his notice at various times since being operated upon.

Dr. Heaton himself gives a large number of cases of his own.

I myself believe all the cases I operated upon, were cured permanently, likewise that this is the very best method now in our hands for treating this difficulty; the pathology is sound, more so than any other operation is based upon, and the results so far as they go prove it and justify a more general and extended application of it. But one case is mentioned by Dr. Heaton when a post mortem was held upon one of his cases, which died some years after being operated upon, and it was found most satisfactory; the hernial passages were completely obliterated, in fact as compared with the sound side it was difficult to understand a hernia had ever existed.

This method is not limited to reducible inguinal hernia alone, but to irreducible as well, and with some modifications to other hernia. Space, and the fact that I am not giving original views of my own, but describing another's and giving my experience, forbid my entering further into the subject. I may say, however, that irreducible hernia are made reducible by breaking up the adhesions by manipulation, subcutaneous section, or in some cases by even opening the sac and the bowel or omentum reduced, the omentum, in some instances, when very large and changed in stricture, being ligated and amputated. Many illustrative cases are given to justify this last procedure, which, in reality, is not so frought with danger as at first would appear. Considering the dangers to which all irreducible hernia are liable, the difficulty of protecting and keeping the protrusion from increasing in size, this operation is of great value.

Dr. W. Dunnett Spanton publishes in the *British Medical Journal*, Dec. 11 and 25, 1880, a paper on this important subject with a new operation of his own, giving 13 cases with 11 cures. His measure proposes to obliterate the hernial passages by means of a cork-screw shaped instrument, which is made to pierce the aponeurosis of the external oblique and conjoined tendon of the internal oblique and transversalis in a rather complicated manner, and brought out in a wound made 2 inches below the spine of the pubis, through which a finger is introduced to locate and guard the various structures and guide the point of the instrument while manipulating. It is left in this position for a week or more, varying with the degree of induration produced. A synopsis of this operation can be seen in the *New York Medical Record* for Feb. 19, 1881. The comparative merits of this and the Heatonian method the reader can draw for himself.

But why operate at all? This will at once be propounded by many conservative practiticners, especially in the light of present medical teaching. There are many and sufficient reasons in my opinion.

A patient with an infirmity of this character is imperfect, and the consciousness of it is, if not always depressing, at least is very annoying. The wearing of a truss is an inconvenience, which many would gladly rid themselves of. Some persons again cannot satisfactorily be fitted with a truss. I know of a case in the practice of a prominent physician here, in Buffalo, where, owing to the presence of a fatty tumor, the proper adjustment of a truss is almost out of the question. A hernia unreduced or irreducible, is liable to many grave accidents, too well known to require mention. A man with this defect, no matter how slight, or what his physical condition otherwise, his social or intellectual standing is debarred from entering the services of the United States, the Army, Navy, Marine Hospital Service, Revenue Marine, etc., which to many is a field where there tastes would lead, and if the physical examination of seaman for the Merchant Marine becomes compulsory, it will prevent many men from shipping and earning their living in that manner. Considering the large number who are defective in this respect, it becomes a serious question, and any reasonable method which can remove it, is to be carefully entertained. In the United States there are about 1 in every 15, who carry a hernia, (Agnew); in France 1 in 13, (Malgaine). Out of 334,321 men examined during the war, 17,296 were rejected, (Agnew), and of all the varieties of hernia, the inguinal was the most frequent.

This is but a meagre contribution wherein I desire to express my views about a method of treating a common infirmity, which at the present time but little is done for its permanent relief. My limited experience has been sufficiently gratifying so far as to warrant my making it known to others who may be interested in the subject. Nor is it without some degree of hesitation that I strongly express them, at variance as they are with the opinions of many authorities and teachers. Yet, as without change of opinion, there is no advancement, I send them out for what they are worth, to take care of themselves, and if they but help to awaken the interest, I believe the subject justifies, their object will be more than accomplished.

